# Bamboo-Based Mesoporous Activated Carbon for High-Power-Density Electric Double-Layer Capacitors

**DOI:** 10.3390/nano11102750

**Published:** 2021-10-17

**Authors:** Ju-Hwan Kim, Hye-Min Lee, Sang-Chul Jung, Dong-Chul Chung, Byung-Joo Kim

**Affiliations:** 1 Research & Development Division, Korea Carbon Industry Promotion Agency, Jeonju 54853, Korea; bear9601@kcarbon.or.kr (J.-H.K.); leehm@kcarbon.or.kr (H.-M.L.); 2 School of Chemical Engineering, Chonbuk National University, Jeonju 54896, Korea; 3 Department of Environmental Engineering, Sunchon National University, Sunchon 57922, Korea; jsc@sunchonm.ac.kr; 4 Department of Organic Materials & Fiber Engineering, Chonbuk National University, Jeonju 54896, Korea; 5 Department of Nano & Advanced Materials Engineering, Jeonju University, Jeonju 55069, Korea

**Keywords:** electric double-layer capacitor, bamboo, phosphoric acid, activated carbon, specific capacitance

## Abstract

Demand for hybrid energy storage systems is growing, but electric double-layer capacitors (EDLCs) have insufficient output characteristics because of the microporous structure of the activated carbon electrode material. Commercially, activated carbon is prepared from coconut shells, which yield an activated carbon material (YP-50F) rich in micropores, whereas mesopores are desired in EDLCs. In this study, we prepared mesoporous activated carbon (PB-AC) using a readily available, environmentally friendly resource: bamboo. Crucially, modification using phosphoric acid and steam activation was carried out, which enabled the tuning of the crystal structure and the pore characteristics of the product. The structural characteristics and textural properties of the PB-AC were determined, and the specific surface area and mesopore volume ratio of the PB-AC product were 960–2700 m^2^/g and 7.5–44.5%, respectively. The high specific surface area and mesopore-rich nature originate from the phosphoric acid treatment. Finally, PB-AC was used as the electrode material in EDLCs, and the specific capacitance was found to be 86.7 F/g for the phosphoric-acid-treated sample steam activated at 900 °C for 60 min; this capacitance is 35% better than that of the commercial YP-50F (64.2 F/g), indicating that bamboo is a suitable material for the production of activated carbon.

## 1. Introduction

Recently, concerns about the global environment have increased, and this has spurred studies into environmentally friendly forms of transport, especially alternatives to vehicles based on the internal combustion engine. These vehicles include electric and fuel cell vehicles and are promising because they do not emit harmful exhaust gases such as NO*_x_*, SO*_x_*, or CO*_x_*, unlike internal combustion engine vehicles that use fossil fuels. Energy storage devices with high energy densities, such as Li-ion batteries (LiBs) [1] and fuel cells [2], are key to the development of this technology but are limited by low power densities [3], which make it challenging for vehicles to adapt to varying power demands [4]. However, a hybrid energy storage system (HESS) is a promising alternative. A HESS comprises an electric double-layer capacitor (EDLC) and a LiB or fuel cell [4,5].

EDLCs have low energy densities but high power densities, fast responses, and long-cycle lives [6]. Therefore, in a HESS, the EDLC acts as an auxiliary power source, supplementing the insufficient output of the main power source, improving the energy efficiency of regenerative braking, and reducing the size of the HESS. Recently, the applications of HESSs were expanded to energy storage and renewable energy systems, but these output characteristics [7,8]. Therefore, to improve the output characteristics of HESSs, research into how to improve both the energy and power densities of EDLCs is required. Because EDLCs store energy by adsorbing electrolyte ions onto an electrode coated with activated carbon, the electrochemical properties such as the energy and power densities of EDLCs are determined by the pore characteristics of the carbon material [9]. Previously, many EDLC studies focused on improving the energy density of these materials, and, as a result, most studies focused on the development of activated carbon with a high specific surface area and a micropore-rich pore structure [10,11,12]. However, the organic electrolyte’s large ion size (e.g., TEA^+^ in PC ≈ 1.36 nm, BF_4_^−^ in PC ≈ 1.40 nm) and high viscosity have poor compatibility with activated carbon composed mostly of micropores (<2 nm) [13]. The result is a large decrease in capacitance retention at high current densities due to slow mass diffusion [14]. In this regard, higher requirements have been put forward to simultaneously meet high energy density and power density, which necessitates not only the capacity of storage of many electrolyte ions, but also a pore structure that can rapidly mass transfer sufficient charges.

Recently, various electrode materials (such as CNT-bridged graphene 3D building blocks [15], 3D activated graphene [16], 3D Carbon Frameworks [17]) for EDLCs were developed to improve the energy and power density of EDLCs. Leng et al. [17] clearly showed that 3D Carbon Frameworks with high specific surface area and high mesopore volume could simultaneously meet energy density and power density. However, to achieve the desired porous structure, the synthetic methods necessarily involved complex pre-synthesis and the hazardous post-removal via acidic washing. Therefore, there is a need for a simple and eco-friendly new activation method with high specific surface area and mesopore ratio for high-performance EDLCs [15,16,17].

The pore characteristics of activated carbon are determined by the activation process, precursor, and carbonization process, in that order [18], and the activation methods can be classified as physical or chemical [18,19,20]. Of these two methods, chemical activation can produce activated carbon with a micropore-rich pore structure and a high specific surface area [19,20] but at high economic cost. On the other hand, compared to chemical activation, physical activation produces activated carbon with a relatively low specific surface area [20,21]. However, physical activation can produce activated carbon with a mesopore-rich pore structure [22,23], which results in an improved power density when applied in EDLCs and has the advantage of low processing costs. Crucially, in physical activation, pores are formed as the carbon precursor is oxidized, and the amorphous phase is oxidized before the crystalline phase [24]. Therefore, in physical activation, the pore characteristics are determined by the crystal structure of the carbon precursor [18].

Coconut shells are a renewable source of carbon, making them an attractive precursor for the preparation of activated carbon with a low ash content [25,26]. In fact, the most widely used commercial activated carbon (YP-50F) for EDLCs is produced from coconut shells via steam activation. However, coconut-shell-derived activated carbon has a micropore-rich pore structure, so it is not suitable for the preparation of high-power EDLCs. As an alternative activated carbon precursor, bamboo can be used because of its rapid growth and high fixed carbon content [27]. However, bamboo has a higher cellulose content than coconut shells [26,28], which results in highly crystalline charcoal, making it difficult to produce activated carbon with a high specific surface area by physical activation. Therefore, to use bamboo as an activated carbon precursor for applications in high-power, high-energy-density EDLCs, a stabilization process for controlling the carbon crystal structure is required.

In this study, bamboo-derived activated carbon having a high specific surface area and mesopore ratio was prepared for high power and energy capacity EDLCs. Specifically, stabilization with phosphoric acid before steam activation was used to achieve a high yield and favorable textural properties (i.e., high specific surface area and mesopore ratio) in the bamboo-derived activated carbon. The effect of phosphoric acid stabilization on the pore development mechanism was studied by observing the textural properties, as well as crystal structure analysis. In addition, we tested the electrochemical performance of the PB-ACs prepared under different activation conditions, focusing on the effects of their pore and structural characteristics.

## 2. Experimental

### 2.1. Sample Preparation

The bamboo was crushed after drying to less than 2% water content and sieved to a particle size fraction of 2–3 cm.

#### 2.1.1. Phosphoric Acid Stabilization

The sorted bamboo was immersed in phosphoric acid (H_3_PO_4_, 85%, Daejung Chemical & Metals Co., Siheung, Korea) and stabilized at 150 °C for 24 h via impregnation treatment. Typically, biomass is decomposed (dehydrated) by phosphoric acid and cross-linked via phosphate groups [29]. The stabilization temperature was set based on the results of our previous study [30]. Then, the stabilized bamboo was washed in boiling distilled water until the pH of the filtrate reached pH 7 to remove the phosphoric acid, and then, the final stabilized bamboo was dried at 100 °C for 24 h. The yield of stabilized bamboo was approximately 50%.

#### 2.1.2. Carbonization

For carbonization, the bamboo was heated to 700 °C under N_2_ flow at a rate of 10 °C/min and then held at this temperature for 1 h. The carbonization yield of the bamboo was approximately 25%.

#### 2.1.3. Steam Activation

The carbonized or stabilized bamboo was placed in an alumina boat, which was then inserted into a self-made cylindrical tubular furnace (SIC heater, length: 1000 mm, diameter: 100 mm). The carbonized or stabilized bamboo was heated to 900 °C under N_2_ flow at a rate of 10 °C/min. The gas flow was then switched to steam at a rate of 0.5 mL/min and held at this temperature for 20–60 min. The sample was then cooled to below 30 °C under N_2_ flow, yielding the activated carbon product. The thus-obtained activated carbon samples are labeled based on the treatment process, for example, PB-H-9-5. Here, PB or CB indicate phosphoric acid stabilization or carbonization, respectively, H indicates the steam (H_2_O) treatment, 9 indicates an activation temperature of 900 °C, and 2, 3, 4, 5, or 6 indicates the activation period, i.e., 20, 30, 40, 50, or 60 min, respectively.

### 2.2. Characterization

The textural properties of the PB-AC were determined using N_2_ adsorption–desorption isotherms obtained at 77 K (BELSORP-max, BEL Japan, Osaka, Japan). Before the observation, the CB-AC and PB-AC samples were placed into a cylindrical cell and degassed overnight while maintaining a residual pressure of less than 10^−3^ bar at 300 °C. The specific surface area (*S*_BET_) was calculated from the isothermal adsorption curves using the Brunauer–Emmett–Teller (BET) equation [31]. The mesopore volumes were calculated using the Barrett–Joyner–Halenda (BJH) equation from the desorption curves [32], and the pore size distribution (PSD) was calculated using non-localized density functional theory (NLDFT) [33]. The differences in the microstructures of the activated carbon samples prepared using different activation times (20–60 min) were identified using X-ray diffractometry (XRD, MiniFlex 600, Rigaku, Tokyo, Japan) with a Cu *K*_α_ source. The XRD patterns were obtained between 3° and 80° in 2*θ* at a scanning speed of 2°/min. The sizes of the crystallites of CB-AC and PB-AC were calculated using the Scherrer equation, as shown in Equation (1) [34].

*L* = *K*λ/*B*cos *θ* (1)

Here, the constant *K* is either 0.91 or 1.84 for calculating the crystallite height (*L*_c_) or crystallite diameter (*L*_a_), respectively, and λ is the wavelength of the X-rays (1.5406 Å, Cu *K*_α_). *B* is the full width at half maximum (FWHM) of the relevant peak in radians.

### 2.3. Electrochemical Tests

Electrodes were made using the prepared PB-AC by mixing PB-AC, a conductive material, and a binder in 84:7:9 mass ratio. The conductive material was carbon black (Super-P, Timcal, Bodio, Switzerland), and the binders were carboxymethyl cellulose (CMC, Dai-Ichi Kogyo Seiyaku Co., Ltd., Kyoto, Japan), styrene–butadiene rubber (SBR, BM400B, Zeon, Tokyo, Japan), and polytetrafluoroethylene (PTFE, 9002-84-0, Sigma Aldrich, ST. Louis, MO, USA). The PB-AC and carbon black were added to the solution containing the binder and dispersed using a planetary centrifugal mixer (AR-100, Thinky Co., Ltd., Tokyo, Japan) for 20 min. The so-obtained slurry was cast immediately on aluminum foil by using a laboratory scale doctor blade coater, whose blade was set at 152 μm. The coated foil was dried overnight in an oven at 150 °C. EDLCs were constructed using CR2032 coin cells. The electrode was punched into round electrodes 12 mm in diameter. Two symmetric electrodes were isolated using cellulose paper (NKK, Kanagawa, Japan). The electrolyte was 1 M tetraethylammonium tetrafluoroborate/propylene carbonate (TEABF_4_/PC). All electrochemical tests were performed at room temperature with a Maccor 4300 battery tester (Maccor Inc., Tulsa, OK, USA) and a VSP electrochemical workstation (Bio-Logic Science Instruments, Grenoble, France). Galvanostatic charge/discharge (GCD) tests were performed at a current density of 0.1–10 A/g from 0.0 to 2.5 V. Cyclic voltammetry (CV) measurements were performed in the same potential range as the GCD tests at scan rates of 5–400 mV/s. The impedance plots were recorded in the frequency range of 10 mHz to 300 kHz. The cells were assessed for their specific capacitance (capacitance per electrode weight), energy density (Wh/kg) and power density (W/kg), which was calculated using only the weight of the active material and the GCD results using Equations (2)–(4).

(2) $$C_{g}=\frac{i\Delta t}{m\Delta V}$$

(3) $$E=\frac{C_{g}\times {\left(\Delta V\right)}^{2}}{2\times 3.6}$$

(4) $$P=\frac{E\times 3600}{\Delta t}$$

Here, *i* is the discharge current (A), Δ*t* is the discharge time (s), *m* is the mass of the electrode (g), and Δ*V* is the potential difference (V).

## 3. Results and Discussion

### 3.1. Adsorption Isotherms and Textural Properties

**Figure 1:**
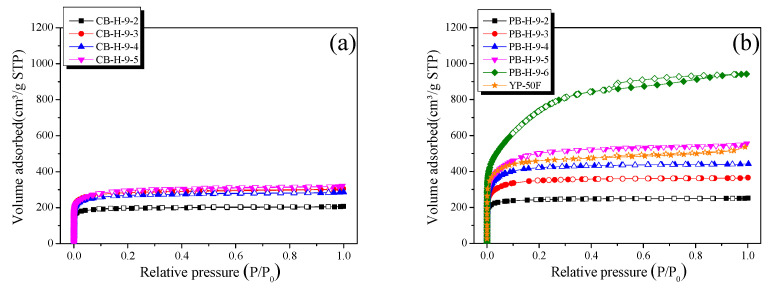
N_2_/77 K isotherm adsorption–desorption curves of (**a**) CB-AC; (**b**) PB-AC.

The N_2_/77 K isothermal adsorption–desorption curve reveals the surface area and pore structure of the activated carbon samples. As shown in Figure 1a, the adsorption–desorption isotherms of CB-AC are type I, according to the IUPAC classification [35]; thus, the pore structure of CB-AC contains many micropores. In addition, an H4-type hysteresis loop, normally associated with slit-shaped pores, was observed for all CB-ACs [35]. The isotherms of the PB-AC samples are shown in Figure 1b and are all type I, indicating a majority of micropores. In addition, we found that a longer activation time is associated with an increased specific surface area and number of mesopores. Therefore, as the activation time increased, the pore structure of PB-AC changed from microporous to mesoporous. In addition, with increase in the activation time, the hysteresis loops of the PB-AC samples increased, and the largest hysteresis loop was observed for the sample steam-activated at 900 °C for 60 min (i.e., PB-H-9-6).

Table 1 lists the textural properties of CB-AC and PB-AC. As the activation time increased, the yields of CB-AC and PB-AC continued to decrease because of crystallite oxidation. In general, the CB-AC samples had lower specific surface areas and total pore volumes than the PB-AC samples prepared under the same activation conditions. The specific surface area and total pore volume of CB-AC were determined to be 770–1120 m^2^/g and 0.32–0.50 cm^3^/g, respectively. As the activation time increased, the micropore volume of CB-AC increased, but only up to a maximum activation time of 30 min, subsequently remaining constant. On the other hand, the mesopore volume of CB-AC increased to 0.03–0.07 cm^3^/g as the activation time increased.

The specific surface area and total pore volume of PB-AC were determined to be 960–2700 m^2^/g and 0.41–1.46 cm^3^/g, respectively. As the activation time increased, the micropore and mesopore volumes of PB-AC continued to increase. Based on the changes in the pore characteristics, further analysis was performed in two distinct stages: (1) stage I, which involves an increase in the number of micropores between activation times of 0–40 min, and (2) stage II, which is characterized by the development of both micropores and mesopores at activation times of 50–60 min. As the activation time increased, the micropore and mesopore volumes of all the PB-ACs increased. In particular, in stage I, PB-AC has a microporous structure because the micropore volume increases more than the mesopore volume. In contrast, in stage II, PB-AC has a mesoporous structure because the mesopore volume increased more than the micropore volume.

**Figure 2:**
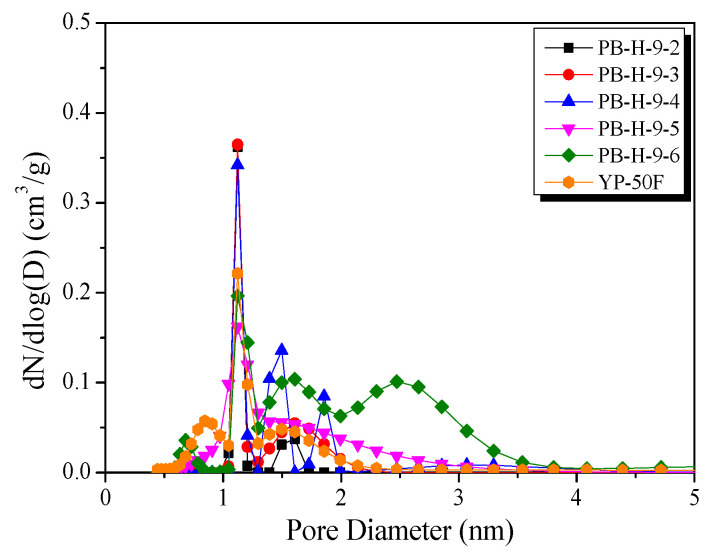
Pore size distributions obtained using the NLDFT method of the bamboo-derived activated carbon as a function of steam activation conditions.

The PSD curves reveal the pore development. Figure 2 shows the PSDs of PB-AC obtained using NLDFT. In stage I, all PB-AC samples have narrow PSDs with pore diameters centered around 1.12 nm; thus, all PB-AC samples have similar pore diameters, but the volume of pores having diameters of 1–2 nm gradually increased with increase in activation time. In stage II, the pore distribution of PB-AC was centered around 1.12 nm but showed a decrease in the volume of pores having a diameter of 2–4 nm. The increase in the pore volume in activated carbon induced by physical activation occurs in two different ways: pore drilling (which results in a steady increase in pore diameter) and pore deepening (which has virtually no effect on pore diameter) [36]. Therefore, in stage I, the micropore volume was increased by pore deepening, and, in stage II, the mesopore volume increased by pore drilling. As shown in Table 1, the textural properties of commercial activated carbon (YP-50F) are similar to those of PB-H-9-5. Further, as shown in Figure 2, YP-50F and PB-H-9-5 have similar PSDs centered around 1.3 nm, but PB-H-9-5 has a higher mesopore volume (≥2 nm) than that of YP-50F.

### 3.2. X-ray Diffraction Analysis

**Figure 3:**
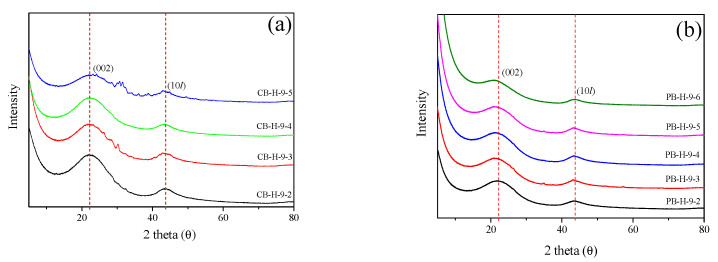
XRD patterns of bamboo-derived activated carbon as a function of various steam activation conditions: (**a**) CB-AC and (**b**) PB-AC.

Figure 3 shows the XRD curves of the bamboo-derived activated carbon. The XRD patterns of CB-AC and PB-AC are typical of isotropic carbon materials, containing peaks corresponding to the C(002) and C(10*l*) planes [37]. As the activation time increased, the intensity of the peaks in the XRD patterns gradually decreased owing due to the oxidation of the crystalline phase. In the XRD pattern of CB-AC, peaks corresponding to ash were observed in addition to those arising from the C(002) and C(10*l*) planes of the carbon. In contrast, in the XRD pattern of PB-AC, only peaks corresponding to the C(002) and C(10*l*) planes of carbon were observed. Naturally derived carbon precursors contain large amounts of ash, but we assumed that the ash content of PB-AC is lower than that of CB-AC because the ash is removed together with monosaccharides (originating from hemicellulose and lignin) during the phosphoric acid stabilization process [38].

**Figure 4:**
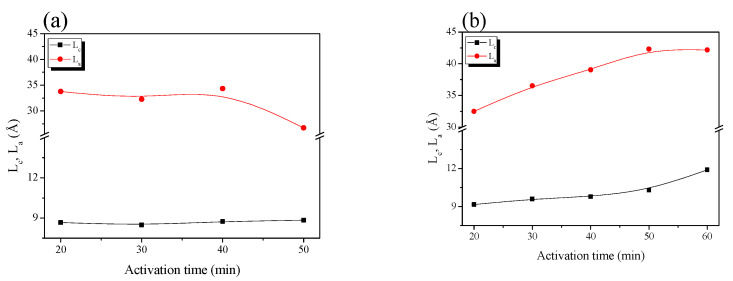
Structural characteristics of the bamboo-derived activated carbon as a function of steam activation conditions: (**a**) CB-AC and (**b**) PB-AC.

Figure 4 shows the crystallite height (L_c_) and size (L_a_) of the bamboo-derived activated carbon. Because the crystal structure of activated carbon is based on an sp^2^ carbon framework, a larger change in L_a_ than L_c_ was observed [24,39]. Further, CB-AC and PB-AC showed distinct changes in the crystal structures with increase in activation time. In the XRD patterns of CB-AC, L_a_ remained unchanged after treatment for 40 min, and L_c_ showed little change with increase in activation time. On the other hand, for PB-AC, both L_a_ and L_c_ increased with an increase in activation time. The difference in pore characteristics between CB-AC and PB-AC is considered to arise from the differences in the oxidation behavior of the crystals. However, it is important to note that XRD data provide information concerning the average crystallite size. Therefore, the increase in structural parameters (L_c_ or L_a_) is considered to be a relative increase arising from the oxidation of amorphous or small crystals rather than crystal growth. On the other hand, the decrease in these structural parameters suggests that more oxidation reactions took place at the edges of the crystalline phase than in the amorphous phase. Therefore, the micropores and mesopores were formed in the activated carbon by the oxidation of amorphous regions or crystalline edges, respectively. Consequently, CB-AC has a lower specific surface area and total pore volume than PB-AC because oxidation mainly occurs at the crystal edges.

The changes to the crystal structure of PB-AC can be divided into two stages with increasing activation time, as previously observed for the textual properties. In stage I, L_a_ increased from 32.5 to 39.0 Å and L_c_ increased slightly from 9.2 to 9.8 Å. However, in stage II, L_a_ remained unchanged, but L_c_ increased from 10.3% to 11.9%. In stage I, the increase in L_a_ of PB-AC can be considered to result from the oxidation of the amorphous region. Specifically, the micropore volume increases because of the oxidation of the amorphous region and small crystallites. In addition, the L_c_ of PB-AC was maintained without significant changes in stage II because the oxidation of the amorphous regions and crystal edges occurred simultaneously. Therefore, in stage II, the micropore and mesopore volumes increased. In conclusion, although CB-AC and PB-AC have the same precursor, their pore development differs because of their different crystal structures resulting from the differences in activation: phosphoric acid treatment or carbonization.

### 3.3. Electrochemical Properties

**Figure 5:**
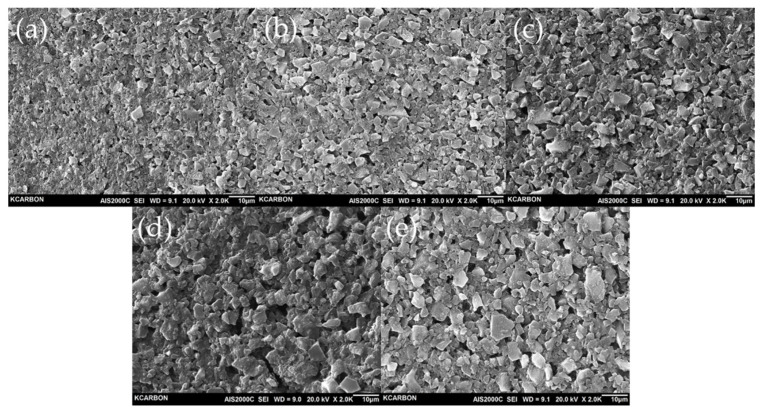
SEM images of the activated carbon electrodes (**a**) PB-H-9-3, (**b**) PB-H-9-4, (**c**) PB-H-9-5, (**d**) PB-H-9-6, and (**e**) YP-50F.

The SEM images were taken to estimate the morphology of the prepared electrode. As shown in Figure 5, the activated carbons are homogeneously distributed with no sign of agglomeration, and signature of electrode cracking was not observed either. Moreover, we did not notice any significant difference between the morphology of the YP-50F electrode and PB-AC electrode, hence, the procedure for electrode preparation with PB-AC is a competitive in that the electrode components are well dispersed. The shorter the activation time, the more relatively densely observed the surface of PB-AC; this was due to the high apparent density.

**Figure 6:**
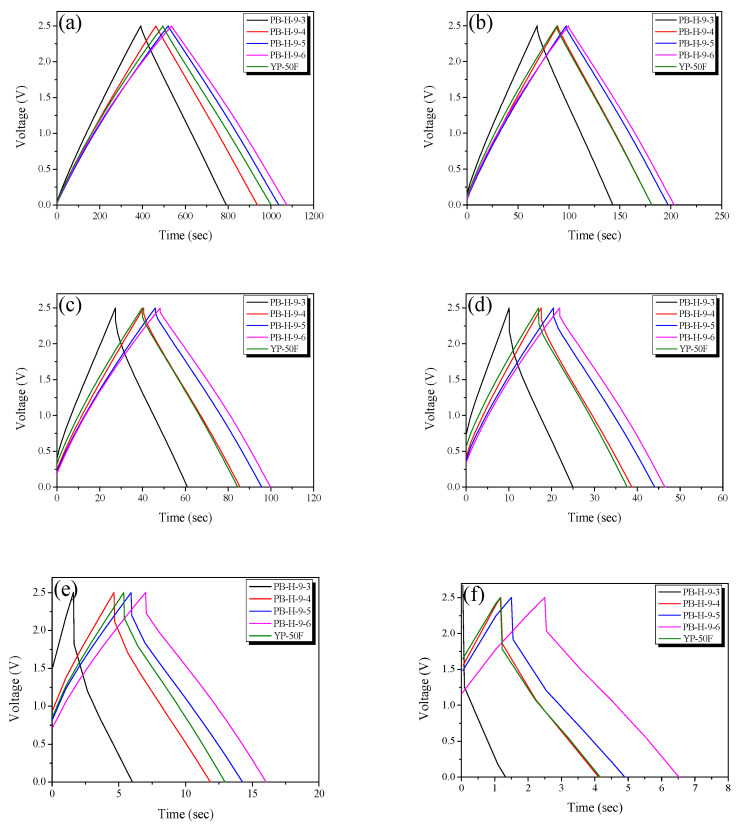
Charge–discharge curves of bamboo-derived activated carbon materials as a function of steam activation conditions obtained at (**a**) 0.1, (**b**) 0.5, (**c**) 1.0, (**d**) 2, (**e**) 5.0, and (**f**) 10.0 mV/s.

The electrochemical properties of the PB-AC electrodes were assessed using GCD, CV, and impedance measurements. The measurements were carried out in 1 M TEABF_4_/PC. Figure 6 shows the GCD curves of the PB-AC electrode at various current densities. The GCD curves of all PB-ACs are linear and symmetrical, which is typical of an ideal EDLC. No obvious *IR* drop was observed in any of the curves at a current density of 0.1 A/g. However, the *IR* drop of PB-AC increased with an increase in current density. The *IR* drop in the GCD represents a voltage change arising from changes in the internal resistance of the electrode, which is related to the self-resistance of the electrode, electrolyte resistance, distance between the electrodes, and contact resistance between the electrode and current collector [6]. Therefore, the *IR* drop increased owing to the increase in resistance as the current density increased. Further, the *IR* drops measured for the different electrodes decreased as the activation time increased, and that of PB-H-9-6 was the smallest of the samples at the same current density. As discussed concerning the pore characteristics, the internal resistance decreases as the pore diameter increases, which occurs with an increase in activation time. In addition, the commercial activated carbon (YP-50F) had a higher *IR* drop than that of PB-H-9-5, despite their similar pore structures, whereas the *IR* drop of PB-H-9-4 was similar.

**Figure 7:**
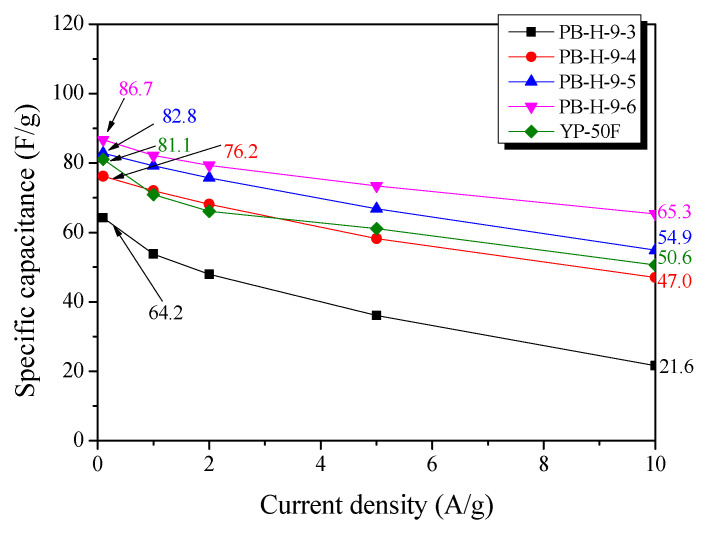
Specific capacitance of PB-AC as a function of discharge current density.

Figure 7 shows the specific capacitances of the PB-AC samples as a function of the current density. As the current density increased from 0.1 to 10 A/g, the specific capacitance decreased owing to the increase in the internal resistance of the PB-AC. Further, the specific capacitance increased with an increase in activation time: 64.2–86.7 F/g at a current density of 0.1 A/g and 21.6–65.3 F/g at 10 A/g. In addition, as the activation time increased, the rate of decrease in the specific capacitance decreased with an increase in the current density. This is presumably because the mesopore volume increased as the activation time increased.

At a current density of 0.1 A/g, YP-50F and PB-H-9-5 have similar specific capacitances, but, at a current density of 10 A/g, the specific capacitance of PB-H-9-5 was approximately 8% higher than that of YP-50F. The changes in the specific capacitance with current density observed for PB-H-9-5 and YP-50F are considered to be closely related to the ion diffusion resistance. PB-H-9-5 has a micropore volume similar to that of YP-50F, but has a higher mesopore volume, and, thus, at a current density of 0.1 A/g, PB-H-9-5 and YP-50F have similar specific capacitances. However, at a current density of 10A/g, PB-H-9-5 has a lower ion diffusion resistance than YP-50F because of its higher mesopore volume. In conclusion, large micropore and mesopore volumes are required to achieve high energy and power densities, respectively, in EDLCs.

**Figure 8:**
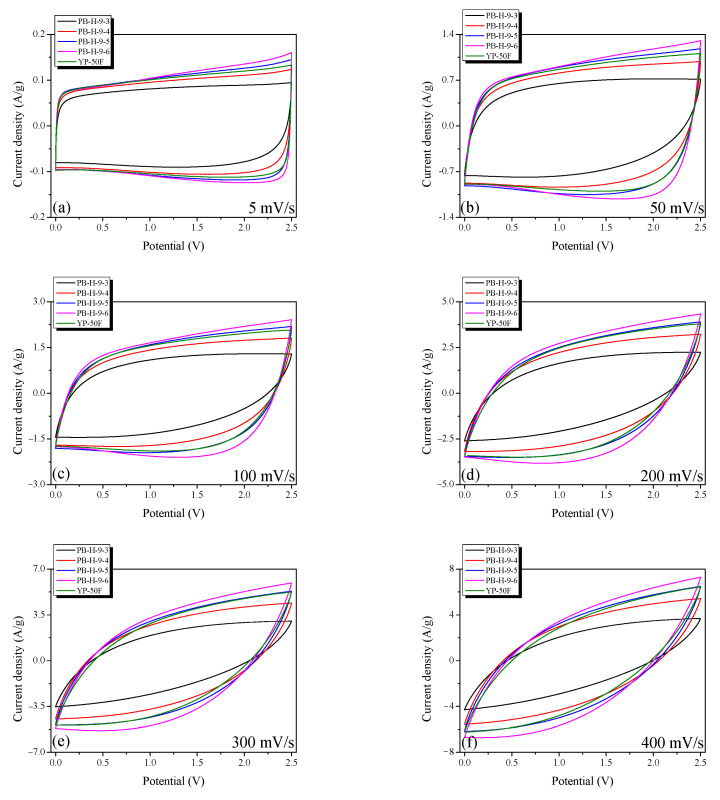
Cycle voltammograms of the bamboo-derived activated carbon samples at various scan rates: (**a**) 5, (**b**) 50, (**c**) 100, (**d**) 200, (**e**) 300, and (**f**) 400 mV/s.

Figure 8 shows the CV curves obtained for the PB-AC electrodes at various scan rates (5–400 mV/s). The current densities in the CV curves are normalized with respect to the mass of the active electrode material. Interestingly, the trends in the CV curves are identical to those observed for the GCD curves in Figure 6. All the electrodes exhibit nearly ideal EDLC behavior, having rectangular CV curves at low scan rates. However, for all electrodes, at high scan rates, the shapes of the CV curves deviate significantly from the ideal rectangular shape. The sizes of the electrolyte ions in 1 M TEABF_4_/PC are 1.35 to 1.40 nm [13]. Thus, the CV curves of PB-H-9-3 to PB-H-9-6 show an ideal rectangular shape at low scan rates because they contain pores with diameters of 1.5–2.0 nm. However, as the scan rate increased, the rectangular CV curve of PB-AC became leaf-shaped because of the increase in internal resistance.

Next, Nyquist plots were prepared to analyze the impedance data obtained for the EDLCs. In a typical Nyquist plot, a semicircle is observed in the high-frequency region and a Warburg line (slope of approximately 45°) is observed in the low-frequency region [40]. The semicircle portion in the Nyquist plot corresponds to the charge transfer process, with the diameter of the semicircle is proportional to the charge transfer resistance (*R*_CT_) [41]. The diffusion coefficient is calculated in the low-frequency region (Warburg impedance) [41]. The diffusion coefficient is related to the mobility of the diffusion ions and is proportional to the squared velocity of diffusing ions, which means that there is faster diffusion of ions with a higher diffusion coefficient [41].

**Figure 9:**
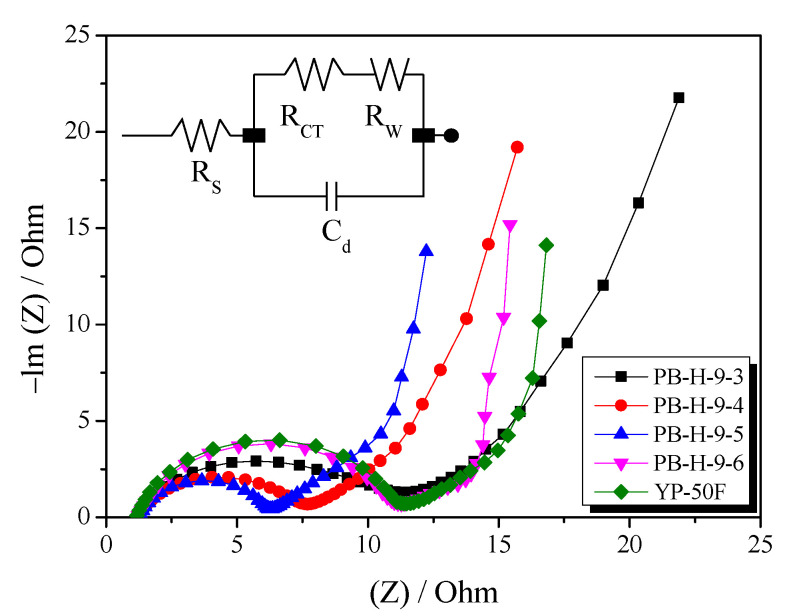
Nyquist plots of bamboo-derived activated carbon materials obtained using different steam activation conditions and its equivalent circuit is shown in inset.

Figure 9 shows the Nyquist plots of the EDLCs prepared containing the PB-AC samples. The data about Nyquist plots were listed in Table 2. As shown in the Figure 8, as the activation time increased, the size of the semicircle decreased until an activation time of 50 min, after which the semicircle size increased. The diameter of the semicircle can be attributed to the interfacial resistance of the electrode pores and the electrolyte. Thus, the interfacial resistance decreased because the pore diameter increased with an increase in activation time (20 to 50 min). On the other hand, the interfacial resistance of PB-H-9-6 increased because of the formation of oxygen functional groups caused by the oxidation of the crystal edges [42].

In the Nyquist plot, the Warburg impedance of the PB-AC appears as a line with a 45° slope and is related to the mass transfer of electrolyte ions. Liu at al. [43] reported that the mesoporous structure of electrode activity material significantly decreases the resistance of EDLC by increasing the diffusion coefficient of ions within electrodes and decreasing the interface resistance. In this study, the mesopore volume ratio of PB-AC increased (44.5%) and the pore diameter increased with increasing activation time. Therefore, the mesoporous structure of PB-AC greatly increases the diffusion coefficient, so that the Warburg impedance is greatly reduced from 4.24 to 2.79 Ω. These results enable PB-AC to have high output characteristics (Figure 8). Although YP-50F has a specific surface area similar to that of PB-AC-H-9-5, it has a low specific capacitance owing to its high impedance.

**Figure 10:**
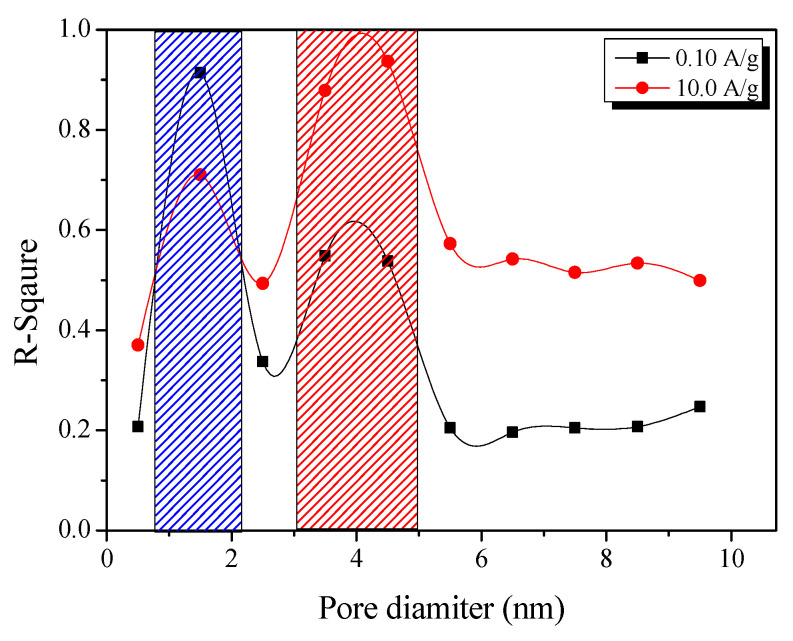
Correlation between the specific capacitance of the PB-AC samples and pore volume. The *x*-axis shows the average PSD plotted as pore volume according to pore diameter in 1 nm units using the average value of each PSD.

The electrochemical behavior of EDLCs is determined by the pore properties of the activated carbon. The crucial role of the pore structure in determining the performance of the EDLCs was confirmed through a correlation analysis between the pore volume, diameter, and specific surface area (Figure 10). At a current density of 0.1 A/g, a coefficient of determination (*R*^2^) of 0.9 was obtained at a pore diameter of 1.5 Å. These results indicate that the specific capacitance of the EDLC is determined by the volume of pores having diameters of 1.5 Å at a current density of 0.1 A/g. As mentioned above, the sizes of the cations and anions of 1 M TEABF_4_/PC electrolyte are 1.35 and 1.40 nm, respectively [13]. Thus, if the volume of pores having a diameter of 1.5 Å is predominant, the area for the adsorption of ions is large, resulting in a high specific capacitance. On the other hand, at a current density of 10 A/g, a broad bimodal PSD in the micropore regions (1.5 nm) and micropore and mesopore regions (widths between 3.5 and 4.5 nm) was obtained (Figure 10). This means that, to achieve a high specific capacitance at a current density of 10 A/g, both the surface area for the adsorption of ions and the diameter of the passages through which ions can move through the micropores without significant resistance are important.

**Figure 11:**
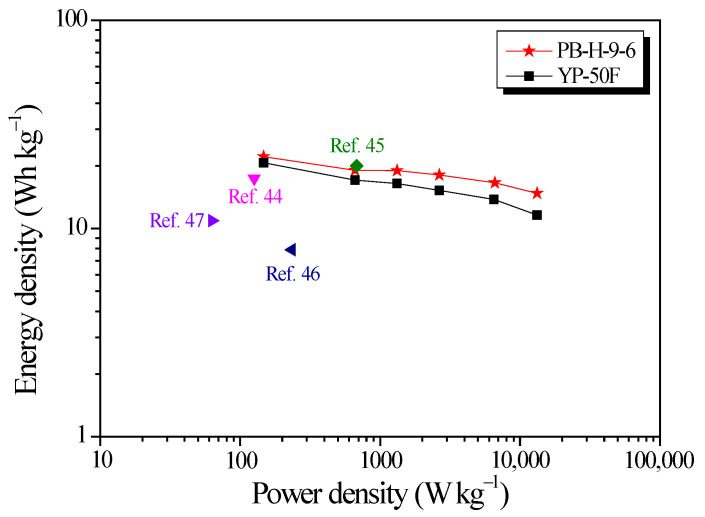
Ragone plot of the bamboo-derived activated carbon device compared with data in other literature studies.

Figure 11 shows the Ragone plot that rendered the relationship of the energy density and power density. The data of Ragone plots are listed Table 3. The EDLC cell based on PB-H-9-6 delivered a maximum energy density of 22.1 Wh/kg at a maximum power density 14.7 W/kg at a current density of 0.1 A/g, outperforming existing data in the literature. Energy density of PB-H-9-6 is higher than that of YP-50F. The relatively small sacrifice in the energy density while increasing the power density is another evidence that PB-H-9-6 has both high specific surface area for ion adsorption and a pore diameter through which ions can move through the micropores without significant resistance. On the basis of the observed results, it can be clearly exhibited that PB-AC is promising for use as electrode materials of high power and energy capacity EDLCs and responsible for ameliorating the forthcoming EDLCs for HESS in an effective way.

## 4. Conclusions

We carried out a comprehensive study of PB-AC, including its textural properties, nanostructure, and electrochemical properties. To prepare the PB-AC, bamboo was stabilized by treatment with phosphoric acid, which resulted in its decomposition, dehydration, and crosslinking. Crucially, the stabilization process yielded a crystal structure different from that produced by carbonization. Thus, based on our findings, bamboo is a suitable precursor for the preparation of activated carbon having a high specific surface area. The specific surface area of the optimal PB-H-9-6 sample was 2700 m^2^/g and had large micropore (0.81 cm^3^/g) and mesopore (0.65 cm^3^/g) volumes. In the activation step, the activation period was varied to control the pore size. As the activation time increased, the pore structure of PB-AC changed from microporous to mesoporous. The microstructure affects the electrochemical performance at a current density of 0.1 A/g. Further, a correlation between the specific capacitance at a current density of 0.1 A/g in 1 M TEABF_4_/PC and the pore characteristics of the PB-AC was determined to result from the matching of the pore size (diameter of 1.5 nm) to the sizes of the electrolyte species. Electrochemical analysis showed that the large mesopore volumes and micropore/mesopore ratios in the PB-AC reduced the ion diffusion resistance, which led to a high specific capacitance when applied in EDLCs at all tested current densities. In conclusion, PB-AC prepared using the phosphoric acid stabilization and steam activation exhibited an enhanced specific surface area and specific capacitance compared to commercial coconut-shell-based activated carbon (YP-50F), which was also prepared by steam activation.

## Figures and Tables

**Table 1:** Textural properties of bamboo-derived activated carbon samples as a function of activation conditions.

Sample	*S*_BET_^a^ (m^2^/g)	*V*_Total_ ^b^ (cm^3^/g)	*V*_Micro_^c^ (cm^3^/g)	*V*_Meso_^d^ (cm^3^/g)	Mesopore Ratio ^e^ (%)	Yield ^f^ (%)
CB-H-9-2	770	0.32	0.29	0.03	9.4	19.6
CB-H-9-3	1100	0.47	0.42	0.05	10.6	13.9
CB-H-9-4	1030	0.44	0.39	0.05	11.4	8.5
CB-H-9-5	1120	0.50	0.43	0.07	14.0	5.8
CB-H-9-6	-	-	-	-	-	0
PB-H-9-2	960	0.41	0.36	0.03	7.7	22.9
PB-H-9-3	1350	0.56	0.51	0.05	8.9	19.4
PB-H-9-4	1630	0.68	0.61	0.07	10.3	16.7
PB-H-9-5	1890	0.86	0.69	0.17	19.8	12.6
PB-H-9-6	2700	1.46	0.81	0.65	44.5	4.6
YP-50F	1780	0.83	0.70	0.13	15.7	-

^a^ *S*_BET:_ The specific surface area; BET method $\frac{P}{v\left(P_{0}-P\right)}=\frac{1}{v_{m}c}+\frac{c-1}{v_{m}c}\frac{p}{P_{0}}$. ^b^ *V*_Total_: Total pore volume; BET method. ^c^ *V*_Micro_: Micropore volume; *V*_Total_ − *V*_Meso_. ^d^ *V*_Meso_: Mesopore volume; BJH method $r_{p}=r_{k}+t$, (*r_p_* = actual radius of the pore, *t* = thickness of the adsorbed film). ^e^ Mesopore ratio: $\frac{V_{\mathrm{Meso}}}{V_{\mathrm{Total}}}\times 100$. ^f^ Yield: $\frac{\mathrm{Weight}\;\mathrm{of}\;\mathrm{activated}\;\mathrm{sample}}{\mathrm{Weight}\;\mathrm{of}\;\mathrm{carbonized}\;\mathrm{or}\;\mathrm{stabilized}\;\mathrm{sample}\;\mathrm{input}\;}\times 100$.

**Table 2:** Values of equivalent circuit parameters from fitting of the impedance spectra in Figure 8.

Sample	*R*_S_^a^ (Ω)	*R*_CT_^b^ (Ω)	*R*_W_^c^ (Ω)
PB-H-9-3	1.26	10.32	4.24
PB-H-9-4	1.24	6.46	3.91
PB-H-9-5	1.25	5.03	3.61
PB-H-9-6	1.22	10.01	2.89
YP-50F	1.18	10.2	4.39

^a^ *R*_s_: bulk electrolyte resistance, ^b^ *R*_CT_: charge transfer resistance, ^c^ *R*_W_: Warburg impedance.

**Table 3:** The electrochemical performance comparison of various biomass-derived activated carbons.

Precursor	Activation Method	*S*_BET_ (m^2^/g)	Electrolyte	Specific Capacitance (F/g)	Energy Density (Wh/kg)	Ref
Baboo	Steam	2700	1 M TEABF4/PC	86.7 @ 0.1 A/g	22.1	This work
Rice straw	KOH	1007	EMIMBF4	64.0 @ 0.1 A/g	17.4	[44]
Corn husk	KOH	1378	1 M TEABF4/AN	64.0 @ 1.0 A/g	20.0	[45]
Stiff silkworm	KOH	2523	6 M KOH	199.8 @ 1.0 A/g	7.9	[46]
Bamboo	KOH	2221	3 M KOH	234.4 @ 0.5 A/g	10.9	[47]

## Data Availability

The data presented in this study are available on request from the corresponding author.

## References

[1] Yu M., Patrick H., von Jouanne A., Yokochi A. (2019). Current Li-Ion Battery Technologies in Electric Vehicles and Opportunities for Advancements. Energies.

[2] Das H.S., Tan C.W., Yatim A.H.M. (2017). Fuel cell hybrid electric vehicles: A review on power conditioning units and topologies. Renew. Sustain. Energy Rev..

[3] Winter M., Brodd R.J. (2004). What Are Batteries, Fuel Cells, and Supercapacitors?. Chem. Rev..

[4] Khaligh A., Li Z. (2010). Battery, Ultracapacitor, Fuel Cell, and Hybrid Energy systems for Electric, Hybrid Electric, Fuel Cell, and Plug-In Hybrid Electric Vehicles: State of the Art. IEEE Trans. Veh. Technol..

[5] Burke A.F. (2007). Batteries and Ultracapacitors for Electric, Hybrid, and Fuel Cell Vehicles. Proc. IEEE.

[6] González A., Goikolea E., Barrena J.A., Mysyk R. (2016). Review on supercapacitors: Technologies and materials. Renew. Sustain. Energy Rev..

[7] Miñambres-Marcos V.M., Guerrero-Martínez M.Á., Barrero-González F., Milanés-Montero M.I. (2017). A Grid Connected Photovoltaic Inverter with Battery-Supercapacitor Hybrid Energy Storage. Sensors.

[8] Sikkabut S., Mungporn P., Ekaravarodome C., Bizon N., Tricoli P., Nahid-Movarakeh B., Pierfederici S., Davat B., Thounthong P. (2016). Control of High-Energy High-Power Densities Storage Devices by Li-ion Battery and Supercapacitor for Fuel Cell/Photovoltaic Hybrid Power Plant for Autonomous System Applications. IEEE Trans. Ind. Appl..

[9] Bang J.H., Lee H.M., An K.H., Kim B.J. (2017). A study on optimal pore development of modified commercial activated carbons for electrode materials of supercapacitors. Appl. Surf..

[10] Karthikeyan K., Amaresh S., Lee S.N., Sun X., Aravindan V., Lee Y.G., Lee Y.S. (2014). Construction of High-Energy-Density Supercapacitors from Pine-Cone-Derived High-Surface-Area Carbons. ChemSusChem.

[11] Phiri J., Dou J., Vuorinen T., Gane P.A.C., Maloney T.C. (2019). Highly Porous Willow Wood-Derived Activated Carbon for High-Performance Supercapacitor Electrodes. J. Am. Chem. Soc..

[12] Tian X., Ma H., Li Z., Yan S., Ma L., Yu F., Wang G., Guo X., Ma Y., Wong C. (2017). Flute type micropores activated carbon from cotton stalk for high performance supercapacitors. J. Power Sources.

[13] Lin R., Taberna P.L., Chmiola J., Guay D., Gogatsi Y., Simon P. (2009). Microelectrode Study of Pore Size, Ion Size, and Solvent Effects on the Charge/Discharge Behavior of Microporous Carbons for Electrical Double-Layer Capacitors. J. Electrochem. Soc..

[14] Li J., Wang N., Tian J., Qian W., Chu W. (2018). Cross-Coupled Macro-Mesoporous Carbon Network toward Record High Energy-Power Density Supercapacitor at 4 V. Adv. Funct. Mater..

[15] Pham D.T., Lee T.H., Luong D.H., Yao F., Ghosh A., Le V.T., Kim T.H., Li B., Chang J., Lee Y.H. (2015). Carbon Nanotube-Bridged Graphene 3D Building Blocks for Ultrafast Compact Supercapacitors. ACS Nano.

[16] Basnayaka P.A., Ram M.K., Stefanakos L., Kumar A. (2013). Graphene/Polypyrrole Nanocomposite as Electrochemical Supercapacitor Electrode: Electrochemical Impedance Studies. Graphene.

[17] Leng C., Zhao Z., Song Y., Sun L., Fan Z., Yang Y., Liu X., Wang X., Qiu J. (2020). 3D Carbon Frameworks for Ultrafast Charge/Discharge Rate Supercapacitors with High Energy-Power Density. Nano-Micro Lett..

[18] Yahya M.A., Qadah Z.A., Ngah C.W.Z. (2015). Agricultural bio-waste materials as potential sustainable precursors used for activated carbon production: A review. Renew. Sustain. Energy Rev..

[19] Mamaní A., Sardella M.F., Giménez M., Deiana C. (2019). Highly microporous carbons from olive tree pruning: Optimization of chemical activation conditions. J. Environ. Chem. Eng..

[20] Nowicki P., Kazmierczak J., Pietrzak R. (2015). Comparison of physicochemical and sorption properties of activated carbons prepared by physical and chemical activation of cherry stones. Powder Technol..

[21] Fu K., Yue Q., Gao B., Sun Y., Zhu L. (2013). Preparation, characterization and application of lignin-based activated carbon from black liquor lignin by steam activation. Chem. Eng. J..

[22] Lee H.M., Heo Y.J., An K.H., Jung S.C., Chung D.C., Park S.J., Kim B.J. (2018). A study on optimal pore range for high pressure hydrogen storage behaviors by porous hard carbon materials prepared from a polymeric precursor. Int. J. Hydrog. Energy.

[23] Lee H.M., Kwac L.K., An K.H., Park S.J., Kim B.J. (2016). Electrochemical behavior of pitch-based activated carbon fibers for electrochemical capacitors. Energy Convers. Manag..

[24] Baek J., Lee H.M., Roh J.S., Lee H.S., Kang H.S., Kim B.J. (2016). Studies on preparation and applications of polymeric precursor-based activated hard carbons: I. Activation mechanism and microstructure analyses. Microporous Mesoporous Mater..

[25] Li W., Yang K., Peng J., Zhang L., Guo S., Xia H. (2008). Effects of carbonization temperatures on characteristics of porosity in coconut shell chars and activated carbons derived from carbonized coconut shell chars. Ind. Crops. Prod..

[26] Daud W.M.A.W., Ali W.S.W. (2004). Comparison on pore development of activated carbon produced from palm shell and coconut shell. Bioresour. Technol..

[27] Choy K.K.H., Barford J.P., McKay G. (2005). Production of activated carbon from bamboo scaffolding waste—process design, evaluation and sensitivity analysis. Chem. Eng. J..

[28] Sugesty S., Kardiansyah T., Hardiani H. (2014). Bamboo as raw materials for dissolving pulp with environmental friendly technology for rayon fiber. Procedia Chem..

[29] Jagtoyen M., Derbyshire F. (1998). Activated carbons from yellow poplar and white oak by H3PO4 Activation. Carbon.

[30] Lee B.H., Lee H.M., Chung D.C., Kim B.J. (2021). Effect of Mesopore Development on Butane Working Capacity of Biomass-Derived Activated Carbon for Automobile Canister. Nanomaterials.

[31] Brauneur S., Emmet P., Telle E. (1938). Adsorption of Gases in Multimolecular Layer. J. Am. Chem. Soc..

[32] Barrett E.P., Joyner L.G., Halenda P.P. (1951). The Determination of Pore Volume and Area Distributions in Porous Substances. I. Computations from Nitrogen Isotherms. J. Am. Chem. Soc..

[33] Kierlik E., Rosinberg M.L. (1990). Free-energy density functional for the inhomogeneous hard-sphere fluid: Application to interfacial adsorption. Phys. Rev. A.

[34] Biscoe J., Warren B.E. (1942). An X-Ray Study of Carbon Black. Int. J. Appl. Phys..

[35] Sing K.S.W. (2009). Reporting Physisorption Data for Gas/Solid Systems with Special Reference to the Determination of Surface Area and Porosity. Pure Appl. Chem..

[36] Wigmans T. (1989). Industrial aspects of production and use of activated carbons. Carbon.

[37] Kim D.W., Kil H.S., Nakabayashi K., Yoon S.H., Miyawaki J. (2017). Structural elucidation of physical and chemical activation mechanisms based on the microdomain structure model. Carbon.

[38] Basta A.H., Fierro V., Saied H., Celzard A. (2011). Effect of deashing rice straws on their derived activated carbons produced by phosphoric acid activation. Biomass Bioenergy.

[39] Baek J., Shin H.S., Chung D.C., Kim B.J. (2017). Studies on the correlation between nanostructure and pore development of polymeric precursor-based activated hard carbons: II. Transmission electron microscopy and Raman spectroscopy studies. J. Ind. Eng. Chem..

[40] Mei B.A., Munteshari O., Lau J., Dunn B., Pilon L. (2018). Physical Interpretations of Nyquist Plots for EDLC Electrodes and Devices. J. Phys. Chem. C.

[41] Choi W., Shin H.C., Kim J.M., Choi J.Y., Yoon W.S. (2020). Modeling and Applications of Electrochemical Impedance Spectroscopy (EIS) for Lithium-ion Batteries. J. Electrochem. Sci. Technol..

[42] Oda H., Yamashita A., Minoura S., Okamoto M., Morimoto T. (2006). Modification of the oxygen-containing functional group on activated carbon fiber in electrodes of an electric double-layer capacitor. J. Power Sources.

[43] Liu X., Juan L., Zhan L., Tang L., Wang Y., Qiao W., Liang X., Ling L. (2010). Effect of conductive filler on the impedance behaviors of activated carbon based electric double layer capacitors. J. Electroanal. Chem..

[44] Sudhan N., Subramani K., Karnan M., IIayaraja N., Sathish M. (2017). Biomass-Derived Activated Porous Carbon from Rice Straw for a High Energy Symmetric Supercapacitor in Aqueous and Non-Aqueous Electrolytes. Energy Fuels.

[45] Rani M.U., Nanaji K., Rao T.N., Deshpande A.S. (2020). Corn husk derived activated carbon with enhanced electrochemical performance for high-voltage supercapacitors. J. Power Sources.

[46] Gong C., Wang X., Ma D., Chen H., Zhang S., Liao Z. (2016). Microporous carbon from a biological waste-stiff silkworm for capacitive energy storage. Electrochim. Acta.

[47] Zhang G., Chen Y., Chen Y., Guo H. (2018). Activated biomass carbon made from bamboo as electrode material for supercapacitors. Mater. Res. Bull..

